# Understanding the burden of unidentified bodies: a systematic review

**DOI:** 10.1007/s00414-023-02968-5

**Published:** 2023-03-02

**Authors:** Kate Megan Reid, Lorna J. Martin, Laura Jane Heathfield

**Affiliations:** Division of Forensic Medicine and Toxicology, Department of Pathology, Faculty of Health Sciences, University of Cape Town, Cape Town, South Africa

**Keywords:** Forensic human identification, Unidentified bodies, Homelessness, Standardisation, Cross-disciplinary approach

## Abstract

While human identification is a crucial aspect of medico-legal investigations, many individuals remain unidentified each year across the world. The burden of unidentified bodies is often referred to when motivating for improved methods of identification, and anatomical teaching, yet the actual burden is somewhat unclear. A systematic literature review was undertaken to identify articles that empirically investigate the number of unidentified bodies experienced. Despite the large number of articles returned, an alarmingly low number (24 articles) provided specific and empirical details on the number of unidentified bodies, demographics and trends thereof. It is possible that this lack of data is due to the variable definition of ‘unidentified’ bodies and the use of alternative terminology such as ‘homelessness’ or ‘unclaimed’ bodies. Nevertheless, the 24 articles provided data for 15 forensic facilities across ten countries of both developed and developing statuses. On average, developing countries experienced more than double (9.56%) the number of unidentified bodies when compared to developed nations (4.40%). While facilities were mandated under different legislations and infrastructures available varied greatly, the most common issue faced is the lack of standardised procedures for forensic human identification. Further to this, the need for investigative databases was highlighted. Through addressing the standardisation of identification procedures and terminology, alongside the appropriate utilisation of existing infrastructure and database creation, the number of unidentified bodies could be significantly reduced globally.

**Supplementary Information:**

The online version contains supplementary material available at 10.1007/s00414-023-02968-5.

## Introduction

When an individual dies of unnatural causes or suddenly, a medico-legal investigation is undertaken [1]. The purpose of such investigations is threefold: (1) determine the cause of death, (2) aid in the criminal investigation of death and (3) identify the deceased [1]. The first two points carry obvious value in the criminal justice sector. However, point 3 is essential for not only criminal justice, but also social justice [2, 3]. Families of the deceased are entitled to bury or cremate their loved one, and in many cultures, this is to show respect to the deceased. Furthermore, identification of the deceased may provide some assistance in avoiding ambiguous loss and acceptance of death to the living community. Administratively, identification is necessary for the completion of a death certificate which is further required for release of life insurance policies and control of assets. Consequently, an unidentified body not only prevents the reunion of the deceased with their loved ones, but also indicates the family and community are unaware of that individual’s death.

Theoretically, in the event of non-decomposed, non-skeletonised human remains, the process of identification may be straightforward if visual recognition by next of kin is possible [1]. This involves the viewing of the decedent by next of kin and confirming the suspected identity. However, this is often a distressing and traumatic event for the next of kin member and consequently is regarded less reliable due to the effects of confirmation bias, whereby an individual looks for evidence to prove the pre-conceived theory (i.e. identity of the deceased) [4]. Furthermore, in some instances where the body has been damaged significantly (burning, decomposition, scavenging, mutilation), visual identification is not possible [1]. In these instances, scientific means of identification (fingerprint analysis, DNA analysis, anthropological assessment) are required [1, 5–8].

The implementation of these scientific methods is often subject to the opinion of the presiding forensic pathologist, mortuary-specific policies and legislation. Despite developments in these alternative methods, not all forensic facilities are able to make use of them due to a lack of resources and the inability to source ante-mortem reference data [9–11]. This unfortunately leaves a portion of decedents remaining unidentified each year, which places strain, both financially and logistically, on state facilities to store and/or bury these remains.

Mazzarelli et al. drew attention to the number of countries facing unidentified bodies [12]. While this article brings attention to the issues at hand, no study to date has empirically reviewed the reported figures of unidentified bodies, nor the methods of forensic human identification utilised at forensic facilities across the world. Consequently, the aim of this systematic review was to determine what the global extent of unidentified bodies is. The objectives were to (i) collate and compare the number of unidentified bodies experienced globally and explore reasons why this differs between countries, (ii) identify common trends or themes noted with respect to unidentified bodies, (iii) assess the utilisation of scientific methods of identification, and (iv) provide commentary on challenges faced.

## Methods

Three databases (PubMed, Scopus and Web of Science) were searched for relevant articles using key word variations of human identification, unidentified or unclaimed bodies/remains (Table 1). The search period used included all articles published in full up to and including 31 January 2022.

**Table 1:** Articles returned using defined search criteria across three databases

Database	Search query	Number of articles returned
PubMed	*((((((‘unidentified bodies’[Text Word]) OR (‘unidentified bodies’[Text Word])) OR (‘unclaimed bodies’[Text Word])) OR (‘unclaimed remains’[Text Word])) OR (‘unidentified human remains’[Text Word])) OR (‘unidentified decedents’[Text Word])) NOT (‘teaching’[Text Word])*	162
Scopus	“Unidentified” AND “human” OR “unclaimed” AND “human identification” OR “Forensic Identification” AND ( LIMIT-TO ( SRCTYPE, “j”)) AND ( LIMIT-TO ( PUBSTAGE, “final”)) AND ( LIMIT-TO ( DOCTYPE, “ar”)) AND ( LIMIT-TO ( LANGUAGE, “English”))	455
Web of Science	(((((ALL = (“unidentified”)) OR ALL = (“unidentified body”)) OR ALL = (“Unidentified bodies”)) AND ALL = (Forensic)) NOT ALL = (Anatomy)) NOT ALL = (microbiology)	475 (18 book chapters)

All articles returned from the above search terms were preliminarily reviewed for inclusion through reading of the title and abstract. If needed, the full article was read as well. Inclusion and exclusion criteria were the following: (i) only original research articles were included; (ii) articles pertaining to identification in the context of human anatomy teaching or bodies donated for science research were excluded; (iii) articles that were not available in English were excluded; and (iv) articles that did not provide data on the number of unidentified bodies were excluded. Following filtering with the above criteria, the reference list of each included article was ‘hand searched’ to include any remaining articles, until saturation was reached.

Hereafter, each paper was read in full, and variables collected were as follows: publication details; country of population group reviewed; years and numbers of cases reviewed; proportion of unidentified bodies recorded; case details for the unidentified decedents (age, biological sex, nature of death); which methods of identification were implemented; and where applicable, if this was successful; and any commentary, made in the research article, on recommendations for improvement.

Article information was exported from the search databases and converted to an Excel 365 (Microsoft Corporation, NM, USA) workbook. All data variables were subsequently added to this workbook and subjected to descriptive statistical analyses. Graphical representation of results was performed using Excel 365 and Prism (GraphPad Software, USA).

## Results

Across all databases, a total of 810 original research articles were returned, of which 24 articles met the inclusion/exclusion criteria (Supplementary Data). It is important to note that many articles referred to unidentified bodies, with it being called a ‘silent mass disaster’ [13] or ‘humanitarian crisis’ [14]; however, empirical data on the number of unidentified bodies and demographics thereof were not actively investigated. Consequently, these articles were used to supplement findings but could not be included in the full data analysis workflow. Due to the small number of articles which met the inclusion criteria, and variability in the datasets presented, data analysis predominantly followed a summative and thematic approach.

**Fig. 1:**
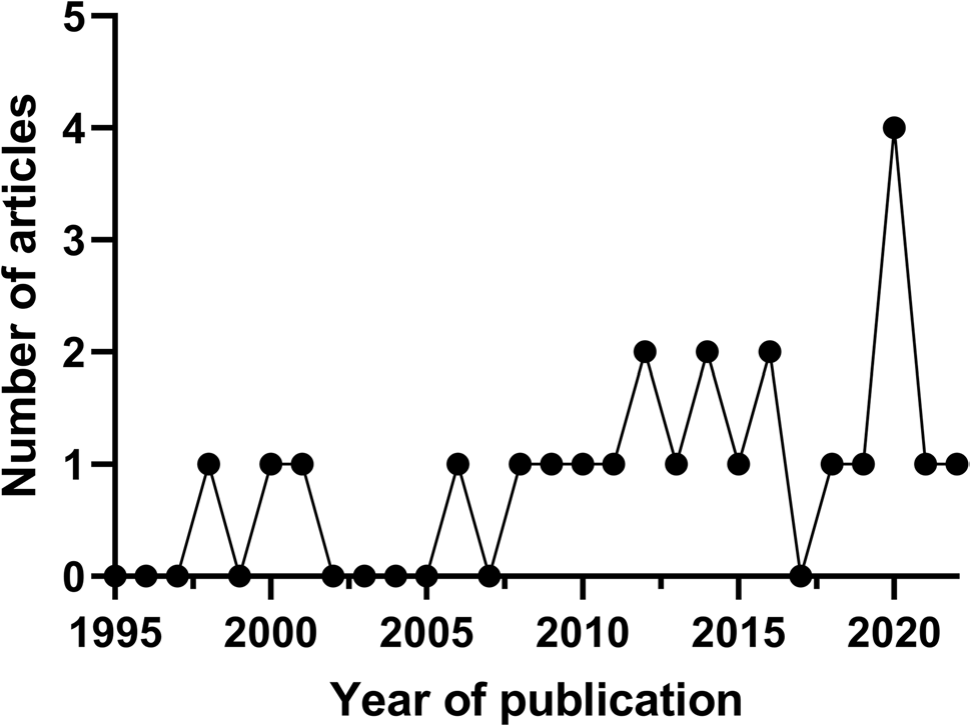
Line plot of number of publications per year

**Fig. 2:**
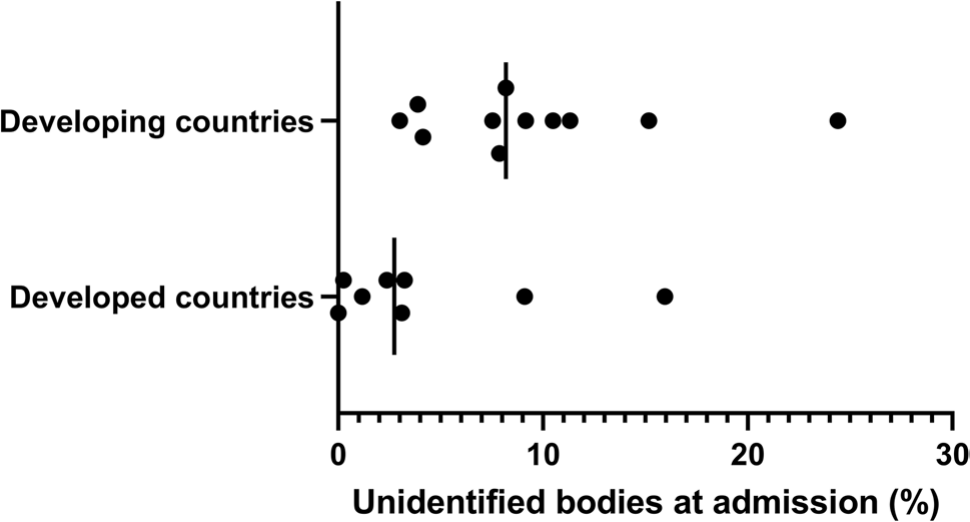
Proportion of unidentified bodies at admission stratified for developing and developed countries

The 24 included articles were published in 15 different journals between 1998 and 2022 (Fig. 1). The articles represented 17 different forensic facilities, spanning 10 countries (Supplementary data). Admission rates of unidentified individuals ranged from < 0.001% [15] to 24.4% [16], and this was largely related to developmental status of a country (Fig. 2). The number of unidentified bodies, experienced at the time of admission, was substantially different between developed (4.401%; range: < 0.001–15.9%) and developing countries (9.56%; range: 3.00–24.5%). Two studies were conducted at the Salt River Mortuary Forensic Facility in Cape Town (South Africa), with a 15-year research gap between the review periods [3, 17]. The average number of annual unidentified bodies increased from 132 (3%; [17]) to 350 (9.2%; [3]) over this time period, despite a relatively stable total case load experienced. Consequently, the former study was excluded from the figures above to provide a more accurate representation of the current situation. Five articles were excluded from these figures as only the number of unidentified bodies was provided and not the proportion of total caseload experienced (Table 2) (Minas Gerias, Brazil: 568 bodies [18]; Jalisco, Mexico: 4060 bodies over 14 years, and estimated 37 433 bodies nationwide [19]; Australia: 500 bodies [20]; Victoria State, Australia: 132 bodies [21]; eight countries in the European Union (EU): 807 bodies [22] and Austria 739 bodies [22]).

**Table 2:** Proportion of cases that were unidentified at admission to a forensic facility and following a full investigation

Reference number	Study location, country	Total case load	Unidentified bodies		Top methods of identification
			At admission	Remaining	
[8]	Georgia, USA	2279	4% (*n* = 100)	0.26% (*n* = 6)	Visual confirmation Fingerprint analysis
[12]	Milano, Italy	22,434	3% (*n* = 726)	1.33% (*n* = 298)	Visual confirmation Fingerprint analysis
[34]	Milano, Italy	14,607	3.1% (*n* = 454)	0.53% (*n* = 77)	Visual confirmation Fingerprint analysis *
[32]	California, USA	683,907	2.37% (*n* = 16,186)	0.40% (*n* = 2752)	Not specified
[16]	Culcutta, India	2515	24.5% (*n* = 614)	20.08% (*n* = 505)	Visual confirmation
[30]	Garches, France	2384	9.1% (*n* = 217)	0.76% (*n* = 18)	Personal items (86.6%) DNA analysis (28.4%)

^*^Different methods were recommended for cases which were poorly preserved

Legislative timelines for the classification of unidentified bodies differed between countries, where the USA [8] and India [11, 16, 23–29] made the official designation after 48 and 72 h respectively, but South Africa did so after 30 days [3]. All articles gave demographic details about the individuals remaining unidentified. The decedent was typically biologically male (average across papers = 77.3%), usually between 30 and 50 years of age. In some articles, a mean age of decedent was provided [11, 18, 23, 26, 28–32], and the average across these ages was 43.28 years. Death was predominantly resultant of natural, accidental, or suicidal manners. Accidental deaths were commonly due to road traffic accidents, while suicidal deaths were mostly as a result of hanging (supplementary data). Bodies were commonly retrieved from railways and roads [11, 16, 24, 26, 28] or from indoor settings (e.g. religious place, household) [21, 29, 31].

Six articles reported on the percentage of individuals that were identified after admission, and these individuals were most commonly identified through visual confirmation or fingerprint analysis (Table 2). Overall, the most common methods of identification utilised were visual confirmation, DNA analyses and fingerprint analysis, although implementation differed depending on the availability of resources, infrastructure and available expertise. The number of cases which were poorly preserved (skeletonised, burnt, decomposed, mutilated) ranged from 0.37% [28] to 100% [33]. At the Institute of Legal Medicine, LABANOF (Milano, Italy) odontology and anthropology were requested when the body was poorly preserved [34], while other authors emphasised the value of genetic and odontology-based investigations in poorly preserved cases [12, 18, 30, 33, 34].

**Fig. 3:**
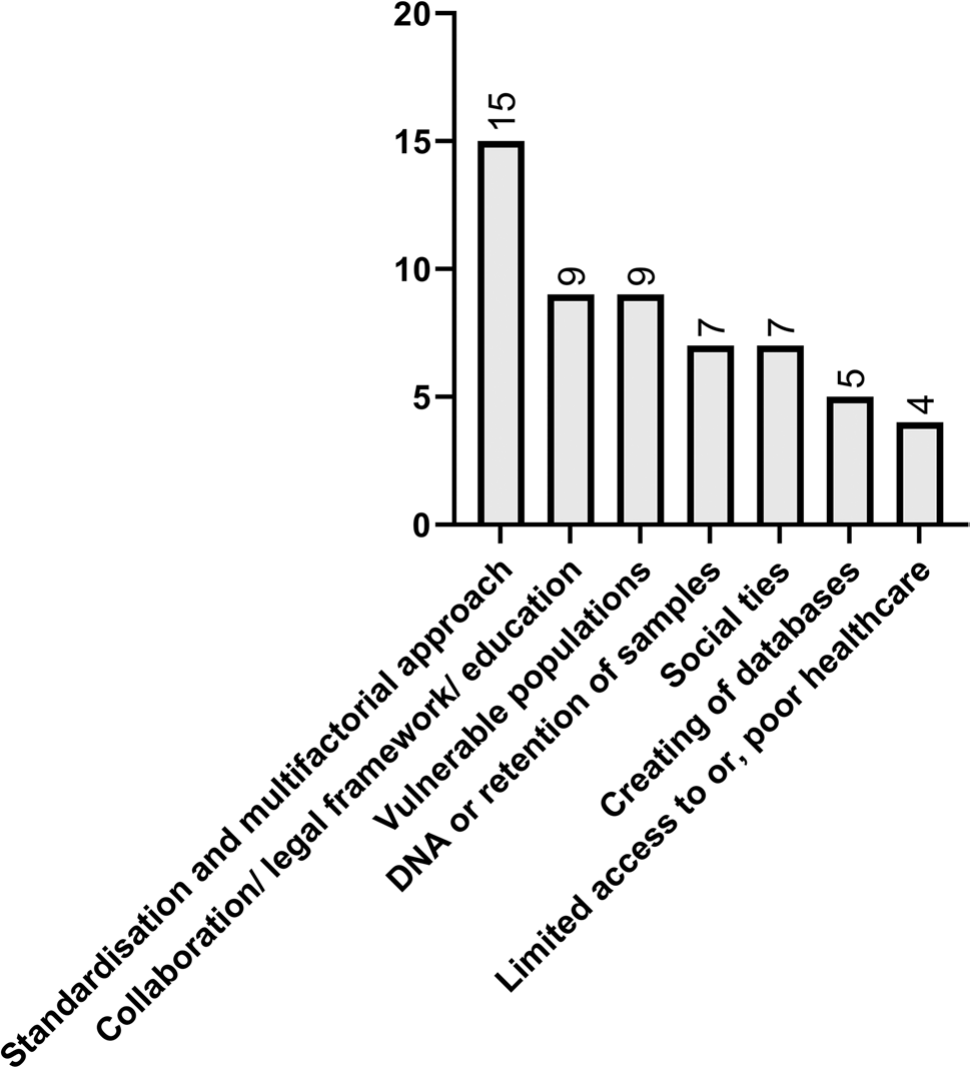
Bar chart indicating key themes used to describe recommendations for improving forensic human identification procedures (numbers represent the number of articles making the recommendation)

Across all research articles included in this study, issues related to the process of human identification and challenges posed from unidentified bodies were highlighted, and recommendations for improvement were suggested (Supplementary Data). These issues and recommendations were similar across all publications and could be classed into different key themes (Fig. 3). The most common recommendation made was the need for a standardised approach to forensic human identification that encompassed all available methods and the appropriate documentation of methodologies implemented [3, 8, 12, 15, 18, 19, 21–23, 25, 27, 30, 32–34]. Vulnerable groups (homeless, migrant, foreign nationals, or those with poor socio-economic background) were identified as individuals more likely to be unidentified [8, 11, 15–17, 26–28, 32]. In particular, many papers specifically mentioned the value of retaining samples for DNA analyses, and consequently, this was presented as a separate theme [8, 11, 20, 23, 26, 28, 30], as well as the need to establish databases for missing persons and unidentified bodies that accessible and shareable across a nation or internationally [3, 20, 22, 28, 29]. Lastly, the poor access to basic healthcare was noted as a potential focus area in order to curb the number of unidentified bodies experienced [11, 26, 28, 31].

## Discussion

Forensic human identification is a key aspect in medico-legal investigations which provides justice in both a social and legal context [1]. Unfortunately, a proportion of decedents are never identified and this places financial, resource-related and infrastructural pressure on the relevant state authorities. The process of human identification is often straightforward, whereby next of kin view the body and confirm a suspected identity of the individual. When this is not possible, the application of scientific means of identification is needed [1].

While the number of unidentified people and the associated issues is acknowledged, empirical data pertaining to these cases is not widely published. This was evident in the number of papers returned on the search parameters implemented which paid attention to the occurrence of unidentified people but did not provide empirical data on the number or demographics of unidentified bodies experienced. These publications predominantly focused on the ethical consideration of using unidentified bodies in an anatomical teaching capacity [35–39], or alternative methods to improve the process of identification [40–45], without providing empirical data on unidentified persons. The fact that unidentified bodies are used for medical teaching (anatomy practicals), or as the rationale for other scientific research (need for improved methodology and identification procedures), clearly highlights that unidentified bodies are a common issue internationally. While not within the scope of this review article, the abundance of literature surrounding the use of unidentified bodies for teaching and research should be further evaluated as it poses significant ethical and legal issues. Although the paucity of publications providing empirical data is noted, little explanation for this is provided. It could be hypothesised that this reflects poor understanding or a lack of urgency regarding unidentified human remains compared to global health issues of the living, or it could be a factor of the complicated and difficult nature of identification as a whole.

### Unidentified bodies: statistics and reasons

This review identified 24 articles that empirically reviewed the problem of unidentified bodies experienced globally (supplementary data). The proportion of unidentified individuals was frequently attributed to the socio-economic status of the country (Fig. 2), whereby developing countries, such as India or South Africa, were inclined to have higher rates of unidentified bodies [3, 10, 16, 23, 24, 26], when compared to developed nations like the USA or European nations [8, 12, 15, 30, 34]. It is important to note that articles reviewed in this study, generally provided data for a specific mortuary or forensic facility. Consequently, generalisable comments about the country were seldom made due to uncertainty surrounding whether the facility was representative of the country’s status or if it was an outlier facility. However, when data was obtained from a number of facilities in a country, or data interpretation of that study commented on a national level, this was factored into the current analysis.

Accidental or unnatural deaths as a result of injury were common, irrespective of socio-economic standing of a nation (Supplementary Data). Deaths by road traffic or railway accidents were common in densely populated areas such as urban areas [8]. The occurrence of motor vehicle accidents may have been exacerbated where there was a lack of law enforcement on the roads, as is common in India and South Africa [3, 10, 29]. Natural causes were largely provided as the nature of death, which is likely attributed to deaths occurring in persons of lower income and social standing, whereby their access to medical care is limited [16, 24, 28, 29, 31].

What was unexpected was that two studies conducted at the Institute of Legal Medicine in Milano, Italy, found that approximately 68% of unidentified cases were devoid of physical alteration to the body (e.g. decomposition, skeletonisation, mutilation) [12, 34], indicating that almost a third of cases were either decomposed, burnt, skeletonised or otherwise altered. This is significantly greater than that noted for developing nations [3, 10, 28, 29]. However, it is possible that the difference is due to a measure of the number of unidentified bodies experienced in the respective countries. The developing nations have a larger number of unidentified persons, and thus, bodies that are poorly preserved make up a small portion of this, though might be similar in number to that seen elsewhere. Furthermore, poorly preserved bodies are likely the reason for no identification at admission in Italy (Institute of Legal Medicine, Milano) but given the availability of resources, the majority of these cases are subsequently identified through scientific methods (Table 2). Nevertheless, the fact that the majority of bodies were well preserved raises the question of why so many cases were unidentified at admission.

As has been mentioned before, in well-preserved cases (e.g. non-decomposed), identification is usually performed through visual confirmation [1]. This was observed during this literature review, where this was the most commonly implemented method as a first port-of-call [3, 8, 12, 16, 27, 30, 34]. However, it was noted that this method may be subject to biases as a result of emotional instability of or trauma experienced by the person(s) performing the confirmation [27, 30]. While a simplistic and cost-effective method for identification, it can be fraught with logistical complications. For visual identification to occur, next of kin are contacted and required to visit the forensic facility [1]. In this review, it was highlighted that individuals without strong social ties or little community involvement were more likely to remain unidentified [24, 32, 34]. This is frequently true of male individuals of working-class age, who leave their families and hometown to seek work in larger cities, which was reflected in this study by the sex (77.3% male) and age (20–50 years) distributions reported (supplementary data). These poor social ties complicate the process of contacting next of kin or even identifying individuals suitably considered as next of kin. This is further hindered by the cost of travel to the forensic facility, which poorer families may not be able to afford, particularly when inter-provincial or state travel is required due to urban migration [3].

Moreover, individuals may be ‘illegally’ residing in the country, as is common in the global migrant crisis [40, 46–49], and therefore, their next of kin may not have appropriate legal documentation to undertake the responsibility of visual identification [49]. Likewise, if next of kin were to come forward, they risk the chance of being deported or facing legal prosecution, which may deter some family members from coming forward at all.

While scientific methods for identification are certainly useful in curbing the burden of unidentified bodies, they are not always feasible or possible. Fingerprint and DNA analysis requires comparison to a relevant reference population database [1]. Odontology and radiology rely on access to ante-mortem data for analysis [50, 51]. Access to antemortem data, or databases may not be in existence in developing countries, nor if family members of the deceased have not reported the individual as missing [9, 52, 53]. Additionally, the performance of scientific methods of identification takes time and expertise, which again is not always feasible in poorer resource-stricken nations, hereby further perpetuating the burden of unidentified bodies encountered.

### Sub-populations and themes associated with unidentified bodies

Throughout the articles reviewed in this study, and many others returned from the search terms, it was clear that homelessness and migration status of an individual were strongly linked with unidentified bodies [11, 15, 16, 24, 26, 28]. It is important to understand that while all these individuals present an unidentified population, each is a separate vulnerable population. From the literature found, it is clear that within unidentified remains, a further classification encompassing the manner in which an individual is unidentified should be included. An example could include distinguishing between (1) unidentified homeless individuals, (2) unidentified migrators and (3) unidentified domestic or routine forensic cases. This type of classification distinguishes between individuals who have relocated to a new area, established themselves a stable lifestyle, but are legal migrants (group 3) and migrants who have demised during the act of migration as is currently experienced on the Mexico border and across the Mediterranean Sea (group 2).

Studies conducted in understanding mortality among unidentified transient or homeless individuals [54–62] showed that death as a result of intoxication or drug use was common as was natural deaths due to untreated medical conditions (e.g. HIV/AIDS [56, 58–60] or infection). Interestingly, history of psychiatric conditions was considered a risk factor for non-identification [31, 56, 63], although not directly found to be a predictor for death in homeless individuals [64]. Furthermore, it was highlighted that the identity of homeless individuals may be suspected through availability of existing records (e.g. treatment or health service records) [57, 62]. This raises concerns that when an individual is suspected to be homeless, and even when an identity is suspected, the full spectrum of available methodologies is not used to obtain a confirmed identity. These points emphasise that perhaps homelessness or poverty is not the cause of unidentified bodies, but rather inappropriate delegation of state resources and a lack of education is. It may also be proposed that if resources were better allocated to basic health care and subsidised shelters [24, 28, 56, 57], the number of unidentified bodies and homeless individuals would not be as problematic.

Similarly, when discussing unidentified bodies, the presence of undocumented or foreign migrants is often raised [48, 49, 65–67]. According to the Missing Migrants Project’s latest figures, since 2014, over 47,000 individuals have died while migrating internationally, and seeking refuge or asylum, with 320 deaths recorded for 2022 already [68]. Kovras and Robins stressed that the number of deaths occurring at sea, particularly with migrants crossing the Mediterranean Ocean, is increasing and well documented [14, 48, 65]; however, the management of these bodies is falling behind [67].

It was further emphasised, when discussing migrant deaths, that one needs to consider the deceased as well as their loved ones [67]. Given the way mass migration, as a result of seeking asylum or refuge, affects established populations, it is reported on and highlighted as a humanitarian crisis through mainstream news outlets. Consequently, it is assumed that more resources are delegated to deaths as a result of migration. However, one study participant, as interviewed by Kovras and Robins, recalled that in one European nation, successful identification of deceased migrants is at a rate of approximately 20%, while for local citizens, it is almost 97% [67]. When considering these figures, in conjunction with assumptions around homelessness and unidentified bodies faced globally, it is clear that appropriate attention or resources are not provided for the identification of vulnerable and minority populations.

Budowle et al. reported that the number of unidentified human remains in the USA has been exacerbated due to the migrant crisis at the Mexico border [69]. It was recommended that the creation of a database for missing persons and unidentified remains is essential to reducing the number of unidentified migrants and human remains [69]. Spradley et al. concluded that a cross-national and collaborative effort to identification necessary to obtain positive identification [49], which supports Nuzzolese’s call for forensic odontology to be used more routinely and for disaster victim identification (DVI) protocols to be reviewed and applied to unidentified remains [40]. This is not dissimilar to recommendations made by authors who focused on unidentified bodies that pass through forensic facilities following death in ‘everyday’ life (supplementary data).

These findings emphasise that each vulnerable group presents different circumstances through which the individual was unidentified, and some unique challenges are encountered. Regardless of this, all unidentified bodies present a challenge to local authorities, significant ethical and social dilemmas and thus should be afforded the same level of attention and granted equal investigative resources.

### Issues and recommendations

In addition to distinguishing between vulnerable groups, throughout this review, it was noted that ‘unidentified’ and ‘unclaimed’ were often used interchangeably when addressing the topic of bodies that do not have a confirmed identity and consequently the burial of removal of the body becomes the responsibility of the state [11, 23, 24, 26, 28, 31, 32]. While this may be acceptable in some countries, it does not hold true for the globe as other countries have defined circumstances under which the different phrases are used. For example, in South Africa, ‘unclaimed’ is used to describe cases where an identity is known but next of kin are financially unable to take responsibility for the burial or cremation of the remains [3], whereas ‘unidentified’ is used to describe cases where no identity is confirmed and thus no next of kin are contactable. Contrastingly, in Marion County, Indiana ‘unclaimed’ describes deceased individuals where no next of kin is known, or the next of kin is known but do not collect the remains [31]. Saurav et al. highlighted that the Delhi Anatomy Act (1953) defines ‘unclaimed’ bodies as the body of a person who dies under specific conditions and has not been claimed by any near relatives or personal friends within 72 h, yet the article refers to ‘unclaimed/unidentified’ bodies which makes the two terms indistinguishable [28]. Furthermore, in some articles, the term ‘homeless’ was used to describe the unidentified population [15, 16, 23, 24, 26, 28]. Caution should be taken when using these terms in place of one another, as it inherently assumes that all unidentified bodies were ‘unidentified’ due to being homeless which is not necessarily a true reflection of society. Rather, authors should carefully define what the terminology means in a local setting, to allow for better understanding and future comparison of data.

As with variation in terminology used, legislative parameters differ between countries and even between states within a country. In this review, the legislative guidance on when to declare a body unidentified ranged from 48 h up to 30 days [3, 8, 23, 24, 26, 28, 31]. Sohn et al. stated that cremated remains were held for up to 3 years for next of kin to claim [32], whereas Hanzlick et al. noted that after 30 days of no confirmation of identity, the case was treated as ‘cold’ [8]. These variations may affect the figures reported for unidentified bodies, as it is not always clear at which point in a timeline the data is obtained from. Future research on the topic of or related to unidentified bodies should specify when the data was collected and acknowledge that variations may exist due to resolution of identity after data collection period.

Despite differences in terminology and time frames, overall findings for the issues encountered with unidentified bodies, homeless individuals and migrants were consistent across the globe (Fig. 3). The need for standardisation, accurate documentation and recording of ante-mortem data was the most common recommendation across all articles included in this study (*n* = 11/17) [8, 12, 15, 23, 28–33, 70]. This is not surprising, as the forensic community continues to argue for better standardisation, which has led to the creation of guidelines and working groups within disciplines (Scientific Working Group for Forensic Anthropology (SWGANTH) and Scientific Working Group on DNA Analysis methods (SWGDAM)) [50, 71] and for the performance of disaster victim identification [72]. It is thus only logical that the process of forensic human identification be guided by a standardised set of instructions on a global level that makes concessions for different levels of infrastructure and utilises all available methods of identification.

Fulton County Medical Examiner’s Office (GA, USA) has researched this extensively over the past decade [73, 74]. While it was found that the existence of an unidentified persons website was not directly credited with solving cases, it had assisted with location of next of kin [73]. The absence of a direct identification may be attributed to the youth (3 years) of the programme at the time of the publication (2008) and overlap with similar purposed databases [73]. Nevertheless, it was advocated as a useful tool for collection and collation of all relevant reports. This study was later developed into the creation of an unidentified decedent reporting system, which is publicly accessible allowing next of kin to search through key findings in efforts to identify their loved one [74]. The need for a centralised standardised database, which allowed for collaboration between stakeholders, was mentioned by a number of authors as well [3, 23, 28, 29].

Working hand in hand with standardisation is the apparent need for better collaboration and education, which was noted in nine articles of both developed and developing status [3, 12, 16, 20–23, 29, 33] (Fig. 3). Through the education and training of professionals involved in forensic human identification, a better understanding of limitations and advantages of different methods would be known, leading to improved utilisation of methods available that are both feasible and applicable. Moreso, educating the public on the importance of forensic human identification and what it entails will hopefully lead to next of kin reporting missing persons more readily and also rectifying misconceptions particularly surrounding DNA evidence.

Retrospective studies have highlighted that the burial or cremation of bodies prior to the retention of samples for DNA or fingerprints analysis limits the ability to identify an individual months or years later [3, 8, 11, 23, 24, 26, 28, 30]. This is particularly concerning when facilities have acknowledged that some loved ones may return years later to claim the remains of the deceased [3, 32]. Sohn et al. noted that over a 3-year period of retention, a total of 17% of cremated remains were claimed by next of kin [32]. The ability to have cremains, claimed after 3 years, stresses the need for the retention of DNA specimens at the time of autopsy for genetic confirmation later on, as well as the need for an accurate and information database of all unidentified remains. In countries where the cost of scientific analysis is too great, it was recommended that specimens or fingerprint pulps be retained in the event that investigations are continued months or years later [11, 23, 24].

## Conclusion

Unidentified bodies, including homeless and migrant remains, place significant pressure on forensic facilities, nations, and non-profit organisations across developing and developed countries. Despite the number of articles (~ 805 returned on search) referring to this humanitarian issue, the small number of articles (24) that actively reviewed this topic is concerning. The small number of articles may be due to misunderstanding or lack of awareness of the seriousness of this global burden. Nonetheless, practical issues that inhibit the ability to identify these bodies were identified and included a lack of resources, improper funding allocations, lack of expertise and large case load. Social issues further reduce identification efforts, as individuals of vulnerable populations are often overlooked, as is seen when considering individuals of poor socio-economic backgrounds, homeless persons or foreign migrants. A key issue highlighted in this study was the lack of consistent terminology or definition of unidentified bodies. It was recommended that future research clearly defines the population in review to reduce ambiguity. Despite these challenges, all articles emphasised the need for a standardised approach to forensic human identification which makes use of multiple methodologies. Together with these standardised guidelines, it is recommended that facilities seek collaborative agreements with forensic service providers who can assist in human identification. Through collaboration and education, databases should be created which allows for cross-search between unidentified bodies and missing persons. The union of these recommendations, with greater acknowledgement of the issues faced, with increased reporting of empirical numbers, will hopefully lead to improved identification and ultimately bury the burden of unidentified human remains.

## Supplementary Information

Below is the link to the electronic supplementary material.

Supplementary file1 (XLSX 24 KB)

## Data Availability

All data generated or analysed during this study are included in this published article (and its supplementary information files).

## References

[1] Saukko P, Knight B (2005) Chapter 3: The establishment of Identify of human remains. In: Saukko P (ed) Knight’s Forensic Pathology, 3rd ed. Arnold: A member of the Hodder Headline Group, London, UK, pp 98–135

[2] Semma Tamayo A (2020). Missing persons and unidentified human remains: the perspective from armed conflict victims exhumed in Granada. Colombia. Forensic Sci Int.

[3] Reid KM, Martin LJ, Heathfield LJ (2020). Bodies without names: a retrospective review of unidentified decedents at Salt River Mortuary, Cape Town, South Africa, 2010–2017. South African Med J.

[4] Byrd JS (2006). Confirmation bias, ethics, and mistakes in forensics. J Forensic Identif.

[5] Haglund WD, Reay DT, Tepper SL (1990) Identification of decomposed human remains by Deoxyribonucleic Acid ( DNA ) Profiling. J Forensic Sci JFSCA 35:724–729 2348185

[6] Austin D, King RE (2016). The biological profile of unidentified human remains in a forensic context. Acad Forensic Pathol.

[7] Schwark T, Heinrich A, Preuße-Prange A, Von Wurmb-Schwark N (2011). Reliable genetic identification of burnt human remains. Forensic Sci Int Genet.

[8] Hanzlick R, Smith GP (2006). Identification of the unidentified deceased: turnaround times, methods, and demographics in Fulton County, Georgia. Am J Forensic Med Pathol.

[9] Baliso A, Finaughty C, Gibbon VE (2019). Identification of the deceased: use of forensic anthropology at Cape Town’s busiest medico-legal laboratory. Forensic Sci Int Reports.

[10] Evert L (2011) Unidentified Bodies in Forensic Pathology Practice in South Africa. MSc Dissertation, University of Pretoria, South Africa

[11] Kumar S, Verma AK, Ali W, Singh US (2015). Homeless and unclaimed persons’ deaths in north India (Jan 2008–Nov 2012): a retrospective study. Med Sci Law.

[12] Mazzarelli D, Milotta L, Franceschetti L (2021). Twenty-five years of unidentified bodies: an account from Milano. Italy. Int J Legal Med.

[13] Ritter N (2007). Missing persons and unidentified remains: the nation’s silent mass disaster. Natl Inst Justice J.

[14] Ellingham STD, Perich P, Tidball-Binz M (2017). The fate of human remains in a maritime context and feasibility for forensic humanitarian action to assist in their recovery and identification. Forensic Sci Int.

[15] Paulozzi LJ, Cox CS, Williams DD, Nolte KB (2008). John and Jane Doe: the epidemiology of unidentified decedents. J Forensic Sci.

[16] Chattopadhyay S, Shee B, Sukul B (2013). Unidentified bodies in autopsy - a disaster in disguise. Egypt J Forensic Sci.

[17] Lerer L, Kugel C (1998). Delays in the identification of non-natural mortality. Am J Forensic Med Pathol.

[18] de e Britto Villalobos MI, Santos AS, Horta MCR (2020). Prevalence of traumatic orofacial and dental injury in unidentified bodies—data from a forensic medicine institute in Brazil. Dent Traumatol.

[19] Birngruber CGG, Martinez Peña EG, Corrales Blanco L (2020). The use of tattoos to identify unknown bodies: experiences from Jalisco, Mexico. Rechtsmedizin.

[20] Ward J (2018). The past, present and future state of missing persons investigations in Australia. Aust J Forensic Sci.

[21] Blau S, Rowbotham SK (2022) Not so simple: understanding the complexities of establishing identity for cases of unidentified human remains in an Australian medico-legal system. Forensic Sci Int 330. 10.1016/j.forsciint.2021.111107 10.1016/j.forsciint.2021.111107 34826760

[22] Cattaneo C, Ritz-Timme S, Schutz HW (2000). Unidentified cadavers and human remains in the EU: an unknown issue. Newletter Int Acad Leg Med.

[23] Kumar A, Harish D, Singh A (2014). Cause of death in “John Doe & Jane Doe”: a 5 year review. J Clin Diagnostic Res.

[24] Kumar A, Lalwani S, Behera C (2009). Deaths of homeless unclaimed persons in south delhi (2001–2005): a retrospective review. Med Sci Law.

[25] Patel DS, Vaghela AC, Shaikh MM (2016). Profile of unidentified dead bodies brought to Mortuary, Civil Hospital, Ahmedabad. J Forensic Med Toxicol.

[26] Raghavendra Babu YP, Joseph N, Kadur K (2012). Mortality among homeless and unclaimed bodies in Mangalore city - an insight. J Forensic Leg Med.

[27] Sakthimani M, Peranantham S (2019). Autopsy based analysis of unidentified dead bodies in a tertiary care hospital of South India. J Indian Acad Forensic Med.

[28] Saurav C, Aayushi G, Behera C (2014). Medico-legal autopsy of 1355 unclaimed dead bodies brought to a tertiary care hospital in Delhi, India (2006–2012). Med Leg J.

[29] Singh P, Aggarwal AD, Aggarwal KK (2012). Unidentified human cadavers: an unaddressed issue. J Forensic Med Toxicol.

[30] Cavard S, Alvarez JC, De Mazancourt P (2011). Forensic and police identification of “X” bodies. A 6-years French experience. Forensic Sci Int.

[31] Quinet K, Nunn S, Ballew A (2016). Who are the unclaimed dead?. J Forensic Sci.

[32] Sohn H, Timmermans S, Prickett PJ (2020). Loneliness in life and in death? Social and demographic patterns of unclaimed deaths. PLoS ONE.

[33] Kringsholm B, Jakobsen J, Sejrsen B, Gregersen M (2001). Unidentified bodies/skulls found in Danish waters in the period 1992–1996. Forensic Sci Int.

[34] Cattaneo C, Porta D, De Angelis D (2010). Unidentified bodies and human remains: an Italian glimpse through a European problem. Forensic Sci Int.

[35] Peeler M (2020). An unexpected education — unclaimed bodies in the anatomy lab. N Engl J Med.

[36] Wilkinson TM (2014). Respect for the dead and the ethics of anatomy. Clin Anat.

[37] Dasgupta N (2004). Unclaimed bodies at the anatomy table. JAMA J Am Med Assoc.

[38] Jones DG, Whitaker MI (2012). Anatomy’s use of unclaimed bodies. Clin Anat.

[39] Gürses İA, Coşkun O, Öztürk A (2018). Current status of cadaver sources in Turkey and a wake-up call for Turkish anatomists. Anat Sci Educ.

[40] Nuzzolese E (2012). Missing people, migrants, identification and human rights. J Forensic Odontostomatol.

[41] Thomson J, Clayton T, Cleary J (2020). An empirical investigation into the effectiveness of genetic genealogy to identify individuals in the UK. Forensic Sci Int Genet.

[42] Kootker LM, von Holstein ICC, Broeders J (2020). Reprint of: The effects of decomposition and environment on antemortem H-Pb-Sr isotope compositions and degradation of human scalp hair: actualistic taphonomic observations. Forensic Sci Int.

[43] De Donno A, Mele F, Baldassarra SL (2020). DNA extraction from sternum bone for identification of a saponified body: use of a modified protocol. Anthropol Anzeiger.

[44] Eck CJ, DiGangi EA, Bethard JD (2019). Assessing the efficacy of isotopic provenancing of human remains in Colombia. Forensic Sci Int.

[45] Parks CL, Monson KL (2018). Recognizability of computer-generated facial approximations in an automated facial recognition context for potential use in unidentified persons data repositories: optimally and operationally modeled conditions. Forensic Sci Int.

[46] Last T, Mirto G, Ulusoy O (2017). Deaths at the borders database: evidence of deceased migrants’ bodies found along the southern external borders of the European Union. J Ethn Migr Stud.

[47] Kobelinsky C (2020). Border beings. Present absences among migrants in the Spanish enclave of Melilla. Death Stud.

[48] Piscitelli V, Iadicicco A, De Angelis D (2016). Italy’s battle to identify dead migrants. Lancet Glob Heal.

[49] Spradley MK, Herrmann NP, Siegert CB, McDaneld CP (2019). Identifying migrant remains in South Texas: policy and practice. Forensic Sci Res.

[50] Scientific Working Group for Forensic Anthropology (SWGANTH) (2010) Personal identification. Issue Date 06/03/2010. Available from http://www.swganth.org/index.html

[51] Avon SL (2004). Forensic odontology: the roles and responsibilities of the dentist. J Can Dent Assoc (Tor).

[52] Duijst W, Passier C, Oude Grotebevelsborg B (2016). Lost and found: the identification process of human bodies recovered from the North Sea. J Forensic Leg Med.

[53] Calacal GC, Delfin FC, Tan MMM (2005). Identification of exhumed remains of fire tragedy victims using conventional methods and autosomal/Y-chromosomal short tandem repeat DNA profiling. Am J Forensic Med Pathol.

[54] Altun G, Yilmaz A, Azmak D (1999). Deaths among homeless people in Istanbul. Forensic Sci Int.

[55] Büyük Y, Üzün I, Eke M, Çetin G (2008). Homeless deaths in Istanbul, Turkey. J Forensic Leg Med.

[56] Baggett TP, Hwang SW, O’Connell JJ (2013). Mortality among homeless adults in Boston: shifts in causes of death over a 15-year period. JAMA Intern Med.

[57] Morrison DS (2009). Homelessness as an independent risk factor for mortality: results from a retrospective cohort study. Int J Epidemiol.

[58] Roy É, Haley N, Leclerc P (2004). Mortality in a cohort of street youth in Montreal. J Am Med Assoc.

[59] Hwang SW, Lebow JM, Bierer MF (1998). Risk factors for death in homeless adults in Boston. Arch Intern Med.

[60] Hwang SW, Orav EJ, O’Connell JJ (1997). Causes of death in homeless adults in Boston. Ann Intern Med.

[61] Hibbs JR, Benner L, Klugman L (1994). Mortality in a cohort of homeless adults in Philadelphia. N Engl J Med.

[62] Hanzlick R, Parrish RG (1993). Deaths among the homeless in Fulton County, GA, 1988–90. Public Health Rep.

[63] Cheung AM, Hwang SW (2004). Risk of death among homeless women: a cohort study and review of the literature. CMAJ.

[64] Nordentoft M (2003) 10 year follow up study of mortality among users of hostels for homeless people in Copenhagen. BMJ 327:81. 10.1136/bmj.327.7406.81 10.1136/bmj.327.7406.81 PMC164916 12855527

[65] Cattaneo C, Tidball Binz M, Penados L (2015). The forgotten tragedy of unidentified dead in the Mediterranean. Forensic Sci Int.

[66] Soler A, Beatrice JS (2018) Expanding the role of forensic anthropology in a humanitarian crisis: an example from the USA-Mexico border. In: Sociopolitics of Migrant Death and Repatriation. Springer International Publishing, Cham, pp 115–128

[67] Kovras I, Robins S (2016). Death as the border: managing missing migrants and unidentified bodies at the EU’s Mediterranean frontier. Polit Geogr.

[68] International Organization for Migration (2022) Deaths during migration recorded since 2014. https://missingmigrants.iom.int/data. Accessed 14 Feb 2022

[69] Budowle B, Bus MM, Josserand MA, Peters DL (2020). A standalone humanitarian DNA identification database system to increase identification of human remains of foreign nationals. Int J Legal Med.

[70] Reid KM, Martin LJ, Heathfield LJ (2019). Evaluation of DNA profiles obtained from deceased individuals at Salt River Mortuary (South Africa). Aust J Forensic Sci.

[71] Scientific Working Group on DNA Analysis Methods (2014) Scientific Working Group on DNA Analysis Methods. Interpretation guidelines for Y-chromosome STR typing. https://www.thermofisher.com/content/dam/LifeTech/Documents/PDFs/SWGDAM_YSTR_Guidelines_APPROVED_01092014_v_02112014_FINAL.pdf. Accessed 12 Feb 2017

[72] INTERPOL (2018) Disaster Victim Identification Guide. Approval Available from https://www.interpol.int/en/How-we-work/Forensics/Disaster-Victim-Identification-DVI

[73] Hanzlick R (2006). Identification of the unidentified deceased and locating next of kin: experience with a UID web site page, Fulton County, Georgia. Am J Forensic Med Pathol.

[74] Hanzlick R, Clark S (2008). The unidentified decedent reporting system: a model national website registry for the unidentified deceased. Am J Forensic Med Pathol.

